# Corrosion Behavior of Shot Peened Ti6Al4V Alloy Fabricated by Conventional and Additive Manufacturing

**DOI:** 10.3390/ma18102274

**Published:** 2025-05-14

**Authors:** Mariusz Walczak, Wojciech Okuniewski, Wojciech J. Nowak, Dariusz Chocyk, Kamil Pasierbiewicz

**Affiliations:** 1 Department of Materials Engineering, Faculty of Mechanical Engineering, Lublin University of Technology, Nadbystrzycka 36D, 20-618 Lublin, Poland; 2 Department of Materials Engineering, Faculty of Mechanical Engineering, Rzeszow University of Technology, al. Powstańców Warszawy 12, 35-959 Rzeszów, Poland; wjnowak@prz.edu.pl; 3 Department of Applied Physics, Faculty of Mechanical Engineering, Lublin University of Technology, Nadbystrzycka 36D, 20-618 Lublin, Poland; d.chocyk@pollub.pl; 4 Faculty of Transport and Computer Science, WSEI University in Lublin, ul. Projektowa 4, 20-209 Lublin, Poland; kamil.pasierbiewicz@wsei.pl

**Keywords:** titanium alloy, additive manufacturing, DMLS, corrosion, electrochemistry

## Abstract

Ti6Al4V titanium alloy is one of the most studied for its properties after additive manufacturing. Due to its widely use in medical applications, its properties are investigated in various aspects of surface layer property improvement and later compared to conventionally manufactured Ti-6Al-4V. In this study, the corrosion behavior in a 0.9% NaCl solution of shot peened Ti-6Al-4V prepared using direct metal laser sintering (DMLS) was examined using corrosion electrochemical testing and compared with conventionally forged titanium alloy. Shot peening was performed on previously polished samples and subsequently treated with the CrNi steel shots. Two sets of peening pressure were selected: 0.3 and 0.4 MPa. X-ray diffraction analysis (XRD), X-ray micro-computed tomography (Micro-CT), scanning electron microscope (SEM) tests with roughness and hardness measurements were used to characterize the samples. The conventional samples were characterized by an α + β structure, while the additive samples had an α’ + β martensitic structure. The obtained results indicate that the corrosion resistance of the conventionally forged Ti-6Al-4V alloy was higher than DMLSed Ti-6Al-4V alloy. The lowest corrosion rates were noted for untreated surfaces of CM/ref and DMLS/ref samples and reached 0.041 and 0.070 µA/cm^2^, respectively. Moreover, the development of the surface has an influence on corrosion behavior. Therefore, increasing pressure results in inferior corrosion resistance. However, better performance for shot peened samples was reported in the low frequency range. This is due to the refinement of the grain acquired after the peening process. All the results obtained, related to the corrosion behavior, were satisfactory enough that the all samples can be characterized as materials suitable for implant applications.

## 1. Introduction

Additive Manufacturing (AM) is currently a popular method because of its unique benefits. Comparing AM to conventional product manufacturing such as casting or forging, the printing process is faster and more accurate than the traditional component manufacturing process, especially with highly complex part geometries [1]. This leads to higher productivity at a lower cost [2]. In the case of forging, there is a requirement for large forging instruments with suitably profiled dies that are costly itself and generate costs associated with for maintenance and replacement [3,4]. 3D printing is insensitive to this due to lack of tooling and on-demand production which overall results in a high flexibility of the process [5,6]. This offers opportunities for its use in a variety of promising applications.

The greatest emphasis of the development of additive technologies can be seen in medical applications [7]. This is due to the ever-increasing demand for metal implants characterized by resistance to mechanical and chemical stresses throughout the implant’s lifetime in the patient’s body [8]. High corrosion resistance and biocompatibility are crucial for proper functioning in the organic environment [9]. Orthopedic surgeons are looking for the most optimal ways to build patient-specific implants to best treat orthopedic diseases [10]. The three major types of biomedical alloys commonly used are stainless steel, Co-Cr-Mo alloys and titanium alloys [11].

Among these, titanium alloys are considered to be the most biocompatible with the human body due to the formation of passive layers on the surface of the material [12]. The layer formed is not completely inert as bone tissue can adhere and grow on the surface of titanium alloys, to the point that titanium-based devices can be completely osseointegrated over time [13]. This ability to accelerate bone tissue regeneration or improvement in adhesion and stabilization is used in various biomedical devices, such as scaffolds, artificial joints, pacemakers, stents etc. [14]. Not only titanium alloys have a good biochemical properties, but also they have high excellent physico-mechanical characteristics such as stiffness very similar to that of human bone, high mechanical properties (lightness, high strength-to-density ratio, corrosion resistance) [15,16].

One such titanium alloy is the Ti-6A-4V alloy. Ti6Al4V is an alpha-beta titanium alloy with 6 wt.% aluminum as the α phase stabilizer and 4 wt.% vanadium as the β phase stabilizer, which maintains a two-phase α + β structure at room temperature [17]. Ti6Al4V alloy is difficult to machine. The deformability of titanium alloys during cold forming is limited, which is why components with complex shapes are preferably formed at elevated temperatures [18]. Therefore, additive manufacturing is clearly a better alternative for the production of complex parts than conventional manufacturing [19,20]. However, fabrication by 3D printing technology, can affect the structural changes of the material and thus the significantly differ properties of conventional alloys compared to printed alloys [21]. This is related to the transformation of β to α’ during laser beam processes such as SLM [22] or DMLS [23]. Such a surface state after 3D printing is not ready for operation, because of various process defect. Porosity [24], residual stresses [25] and microstructure defects [26] are some of the defects that are sought to be removed in the post-processing as it affects the surface layer properties including corrosion resistance [27].

Many studies have already compared the corrosion resistance of traditional and printed titanium alloy and indicate the inferior properties of printed alloys. Huang et al. [28] showed that the corrosion resistance of Ti6Al4V in a 0.1 mol/L NaOH solution of a commercially forged sample was significantly better than that of a sample made by SLM. Dai et al. [29], on the other hand, investigated the corrosion resistance in a 3.5% NaCl solution in which he also showed that the sample made of Ti6Al4V by the conventional method had better properties than the SLM-printed sample.

A remedy for improving properties can be the use of surface finishing in the form of shot peening technology. It is based on the application of shots by means of a mechanical surface treatment involving repeated high-velocity shot impact, which creates plastic deformations that removes defects in the surface layer [30]. Avcu et al. [31] investigated the corrosion behavior for shot peened Ti6Al4V titanium alloy produced by pressure-assisted sintering. The corrosion resistance of the alloy was enhanced by the formation of a passive layer, which formed faster after shot peening treatment. However, the use of this technology is not automatically associated with improved corrosion behavior. In the case of Vella et al. [32] the use of a high peening pressure of 0.7 MPa and a large peening medium resulted in a lack of improvement. This was due obtainment of the high surface roughness, which significantly affects the corrosion resistance.

Therefore, it is important to select the right parameters to obtain a properly finished surface layer. This subject has already been approached in previous studies of our research group on Ti6Al4V alloy and corrosion properties [33,34]. In this studies, at different pressure parameters (0.2; 0.3; 0.4 MPa), the DMLSed surface of Ti-6Al-4V was shot peened with different shots (steel shots, ceramic beads, granules of nuts). Good performance results were obtained on surfaces shot peened with CrNi steel shot with 0.3 and 0.4 MPa. Considering the sufficient results obtained for cytotoxicity after 168 h of extraction for human neuroblastoma SH-SY5Y (ATCC, USA) cell and human skin fibroblast BJ (ATCC, USA) cell lines at 72.40% [35] and 87.95%, respectively [36], this medium can be considered as a compromise solution between mechanical and operational properties and cytotoxicity. This research is intended to extend the state of the art related to the corrosion behavior of additively and conventionally produced Ti6Al4V titanium alloy after the shot peening process. In previous studies, we performed the peening treatment right after printing process. In this research, the conventional and additively manufactured specimens will be polished before shot peening process in order to remove the defected surface layer, subsequently treated with CrNi steel shots at different pressure parameters (0.3; 0.4 MPa).

The purpose of this work is to evaluate the corrosion behavior by means of a comparative analysis of a Ti6Al4V alloy produced by direct metal laser sintering (DMLS) and its conventionally made counterpart after a shot peening treatment of pre-polished surfaces with different peening pressure.

## 2. Materials and Methods

### 2.1. Manufacture of Test Samples

**Figure 1:**
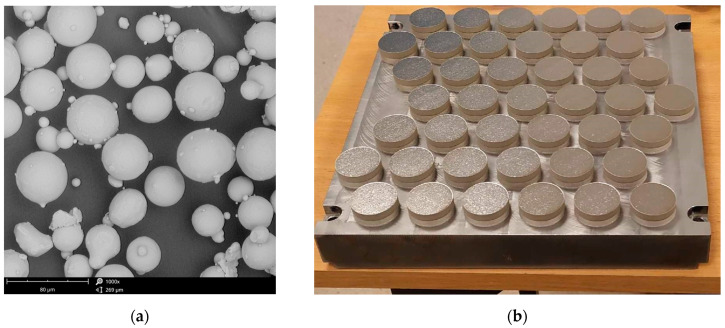
Fabrication of Ti-6Al-4V specimens (**a**) SEM micrograph of Ti-6Al-4V powder; (**b**) Matrix with titanium samples printed using DMLS technology.

In order to investigate the corrosion behavior of shot peened Ti6Al4V alloy fabricated by conventional and additive manufacturing, electrochemical corrosion experiments have been carried out. EOS GmbH EOSINT M280 printer (Krailling, Germany) was used for the fabrication of the AM specimens with a 30 mm diameter and height of 6 mm employing gas-atomized powder with powder particle size not exceeding 63 µm for 99.7 wt %. Ti6Al4V particles used in this process are shown in Figure 1a.

The thickness of the single layer was set to 30 µm. The sintering process was performed using a 200 W laser in an argon protective atmosphere with the laser exposure speed of 1250 mm/s and the laser beam diameter of 100 µm as suggested by the EOS manufacturer standard specifications. The first 3 mm of the print was a dense support structure in the form of a square mesh with 80% fill. After manufacturing, the samples attached to the building plate (Figure 1b) made of Ti6Al4V were separated using a belt cutter.

The other group of the specimens was fabricated from annealed wrought bars from which discs were cut with 30 mm in diameter and 5 mm in height. The declared chemical compositions of both materials were similar and compliant with ASTM F1472, ASTM F2924 (Table 1) [37,38].

The titanium discs as produced were subjected to a grinding procedure. Water-based abrasive papers in grits of 300, 600, 800 and 1200 were used. The grinding procedure was carried out on an ATM SHAPIR 330 (Mammelzen, Germany) grinder-polisher.

### 2.2. Shot Peening Treatment

**Figure 2:**
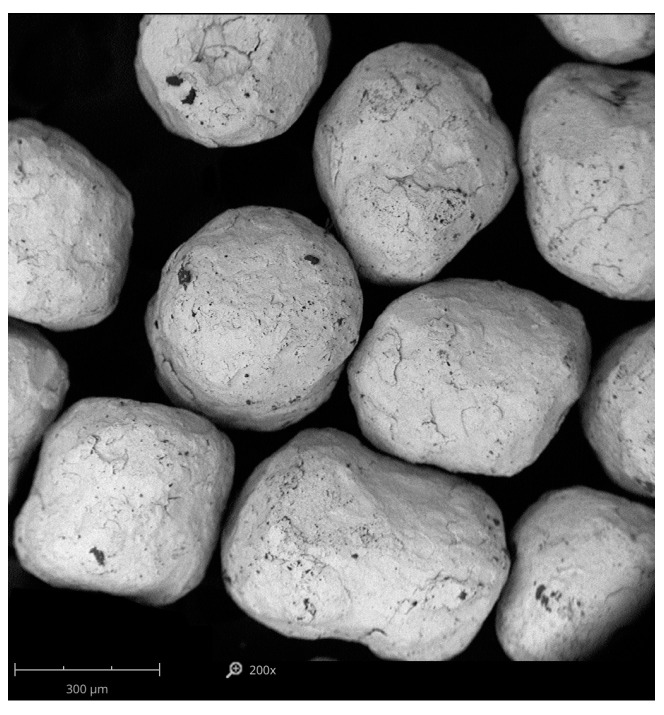
SEM micrograph of peening medium of stainless steel shot CrNi.

The specimens have been subjected to shot peening treatment with IEPCO’s Peenmatic micro 750S device (Leuggern, Switzerland). The process parameters applied were as follows: the peening pressure of 0.3 and 0.4 MPa, peening time of 120 s. Steel shots were deployed during the process. The shot characteristics, together with micrographs are shown in Table 2 and Figure 2.

The products obtained directly after manufacturing (without surface modification) have been used as reference surfaces. Specimen notations are presented in Table 3.

### 2.3. Characterization of Surface Morphology

Two types of microscope were used to analyse the surface morphology characteristics: an optical microscope, MA200 (Nikon), for comparative analysis of the surface microstructure of AM and CM samples, and a nanoscanning electron microscope, Phenom Pro X (Phenom-World, Waltham, MA, USA), for analysis of the surface after shot peening treatment. For SEM images the Back Scatter Detector (BSD) detector and topographic mode was used. Surface analysis was performed at 500× and 1000× magnifications.

### 2.4. Porosity Determination

In order to measure the pore characteristics of the fabricated samples, X-ray micro-computed tomography-microCT Xradia 510 Versa (Carl Zeiss X-Ray Microscopy, Inc., Dublin, CA, USA) with a resolution of 1 µm was adopted. The 1601-element image set was generated using the Reconstructor software 16.1.13038 (Carl Zeiss X-Ray Microscopy, Inc., Dublin, CA, USA) and then reconstructed into cross-sectional images of the scaffolds. Total porosity and pore diameters were determined using CTAnalyser software 1.23.02 (Bruker MicroCT, Kontich, Belgium).

### 2.5. X-Ray Analysis

X-ray diffraction (XRD) analysis was performed using a high-resolution diffractometer (XRD, Empyrean, Panalytical, Minato City, Tokyo) with Cu K-α radiation and a Ni filter. The parameters used for the analysis were voltage of 40 kV, current of 30 mA and a room temperature. All measurements were made using Bragg-Brentano geometry from 2θ = 20° to 100° with a step of 0.01° and a counting time of 6 s per data point. The software utilized for analysis was High Score Plus software package (version 3.0e, Malvern Panalitical, Malvern, UK).

### 2.6. Measurements of Roughness and Hardness

Surface roughness characterization was conducted using a contact profilometer (Dektak 150, Veeco, Plainview, NY, USA). For each tested surface, 5 measurements of the following roughness parameters were made on a measuring distance of L = 5 mm. Changes in hardness of the modified surfaces were examined using a Vickers FM-800 micro-hardness tester (Future-Tech Corp., Kawasaki, Japan). Measurements were made using a load of 200 g (HV0.2) and a measurement time of 15 s. Ten imprints were made for each of the tested surfaces.

### 2.7. Corrosion Tests

In order to determine the corrosion behavior by means electrode impedance spectroscopy measurements in 0.9% NaCl solution, ATLAS 0531 set was used. EIS data acquisition began after stabilization of the sample for 30 min in an open circuit OCP. The circuit consisted of three electrodes i.e., a platinum electrode was used as the control electrode and the reference electrode was a SCE (saturated calomel electrode). Frequencies were scanned between 100 kHz ÷ 100 mHz with 7 points per frequency decade, and with 10 mV sinusoidal signal amplitude. The exposed areas of the working electrode was 0.5 cm^2^.

Subsequently, accelerated corrosion tests were performed in a corrosive medium of 0.9% NaCl on an Atlas Solich ATLAS 0531 Electrochemical Unit & Inpedance Analyser (Poland, Gdańsk). Corrosion tests in the voltage change range of −0.5 V to +1.6 V (potential change rate of 1 mV/s), The results from the electrochemical measurements were processed in AtlasCorr software v.1.04. The values of parameters such as current density of the corrosion i_corr_ and corrosion potentials E_corr_ have been estimated from Tafel curves thanks to potentiodynamic curves analysis in AtlasLab program v.2.29.

## 3. Results and Discussion

### 3.1. Characteristics of the Fabricated Samples

#### 3.1.1. Density and Pore Characteristics

Conventionally produced Ti6Al4V samples have a solid structure. With 3D printing, however, the resulting structure can be a porous structure. Porous materials demonstrate different operational and mechanical properties. Pałka et al. [39] distinguish between two types of porosity open and closed. Open porosity can have a beneficial effect on material properties such as permeability or corrosion resistance, where material with higher porosity shows less susceptibility to corrosion [40]. Voids in medical applications, such as scaffolds, act as channels for filopodia proliferation, vascularization and the osteointegration process, which improves the biocompatibility of the implant [41]. On the other hand, the relatively small pores present in the cell walls favor electrolyte placement and oxygen depletion, so important for the stability and preservation of the oxide layer on titanium, resulting in an increased susceptibility of porous materials to local corrosion [42].

**Figure 3:**
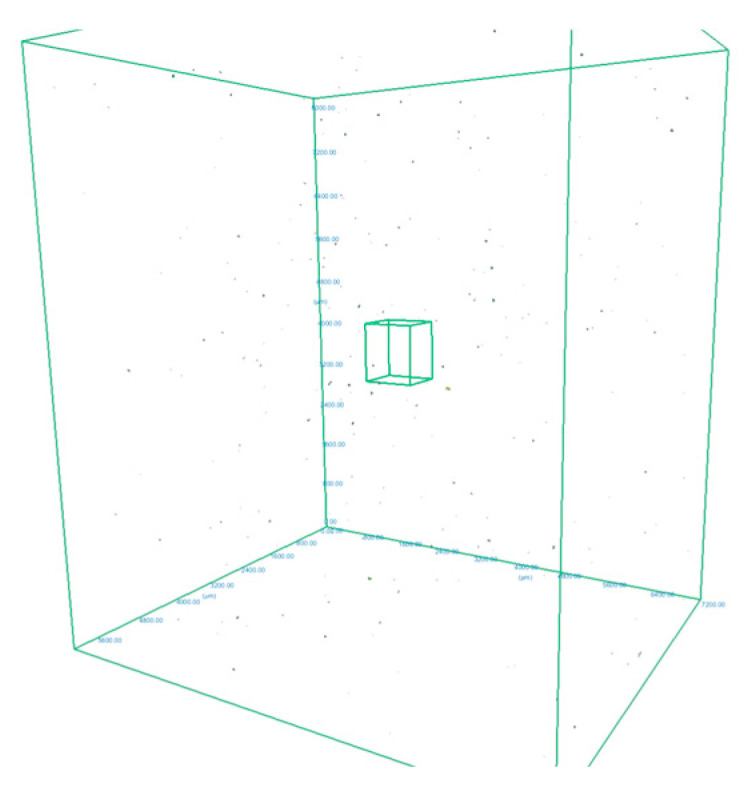
The 3D model of an AM sample obtained from the Micro-CT with pore images and highlighting of the analysed cube.

**Figure 4:**
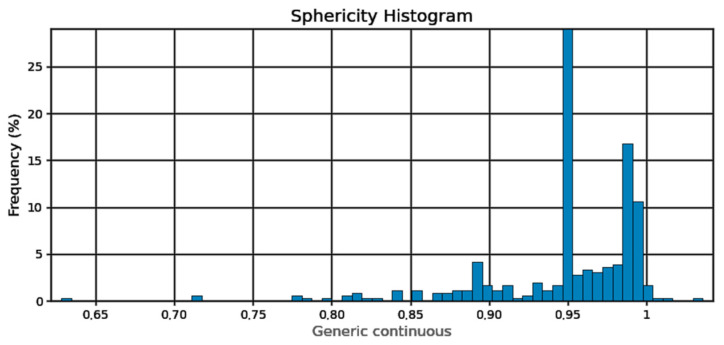
Pore sphericity histogram.

**Figure 5:**
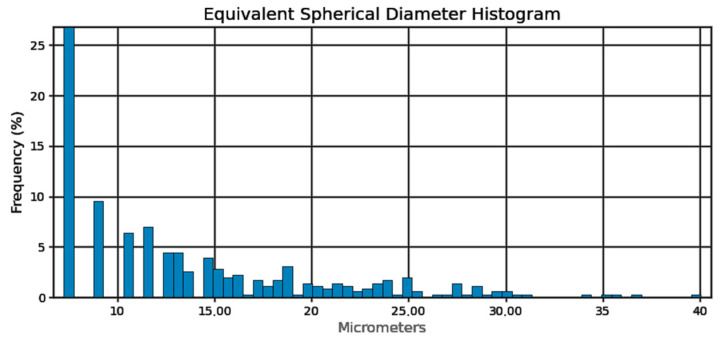
Pore sphericity diameter equivalent histogram.

**Figure 6:**
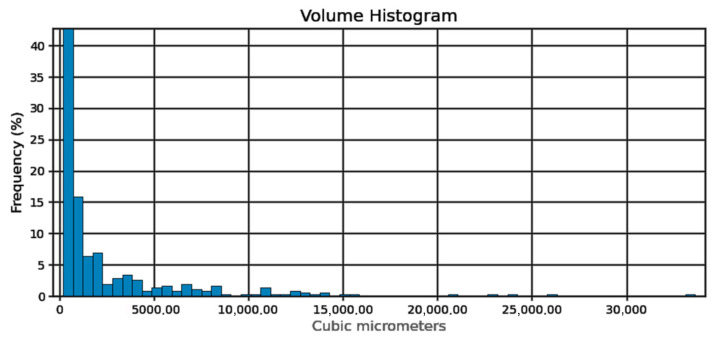
Pore volume histogram.

In order to measure the pore characteristics of the fabricated samples, the analysis was conducted on a selected cube from generated 3D model, measuring 6.1 × 7.2 × 8.1 mm which green contour is visible in Figure 3. The white area represents the material while the dark spots represents the pores detected by the micro-CT. The basic pore characteristics (sphericity, spherical diameter, volume in the form of histograms) are shown in Figure 4, Figure 5 and Figure 6:

On the basis of the designated cube, 358 pores were detected. This represents a porosity of 0.0003%, which implies relative density of Ti-6Al-4V at 99.9997%. This was calculated using the Equation (1), based on the definition [43]:

(1) $$\theta =\frac{V_{p}}{V_{t}}\cdot 100\%$$

where:

θ—porosity, V_p_—pore volume (cm^3^), V_t_—total volume of the object (cm^3^)

Sphericity of the pores (Figure 4) can be determined at 95%. From the pore diameter equivalent histogram (Figure 5), majority of the pores were smaller than 5 μm in size with a volume (Figure 6) of no more than 2500 cubic micrometers.

The number of detected pores is suspected to be lower than expected as capability of the micro-CT depends on the voxel size. Voxel size is determined by the size of the specimen and the capacity of the machine. In this study, a voxel resolution of 1 μm was used, which indicated that pores smaller than approximately 7.22 μm may not have been detected. Relative density at 99.99% was reported by Ratanapongpien et al. [44] in their study of the effect of laser scanning speed and fine shot peening on pore characteristics of L-PBF of Ti-6Al-4V using Micro-CT.

There are many indications in the literature [45,46] of a correlation between porosity and processing parameters described by energy density in TiAl6V4 produced by selective laser melting. The Equation (2) showing the energy density E_v_ is as follows:

(2) $$E_{V}=\frac{P}{v\cdot \delta \cdot h}[J\cdot {mm}^{-3}]$$

where:

P—laser power [W], v—scanning velocity [mm∙s^−1^], h—hatch distance [µm] and δ—layer thickness [µm]

For our samples Volumetric Energy Density is 80 J ∙ mm^−3^

Kasperovich [47] demonstrated that the porosity of additively manufactured parts can be significantly reduced with well-optimized process parameters to a residual porosity of <0.05%, which makes the effect of porosity on corrosion resistance negligible.

#### 3.1.2. Evaluation of the Morphology of the Ti-6Al-4V Phase Components on AM and CM Specimens

**Figure 7:**
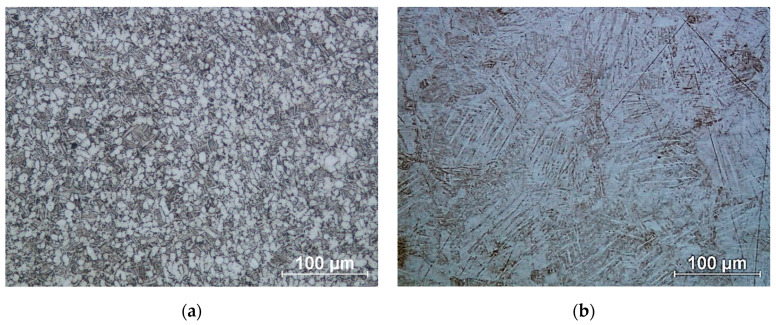
Microstructure of titanium alloy Ti-6Al-4V: (**a**) Wrought bar; (**b**) DMLS printed.

Using an optical microscope, the morphology of the fabricated titanium discs was examined. Photographs of the microstructures are shown in Figure 7.

In the case of the conventional structure, a transformed β-phase is released during cooling, which can be seen as dark areas in Figure 7a. These are lamellar α-phase separations in the β-phase matrix. In contrast, the white areas are equiaxial grains of the α phase [48]. In the case of the additive structure (Figure 7b), we are referring to the martensitic structure of the Ti-6Al-4V alloy. The martensitic microstructure formed has a similar α’ martensite lamellar morphology, but different size and orientation [49]. The structures thus obtained have different corrosion resistance properties [50].

### 3.2. Analysis of Characteristics of Ti-6Al-4V Peened Specimens

#### 3.2.1. XRD Phase Analysis

**Figure 8:**
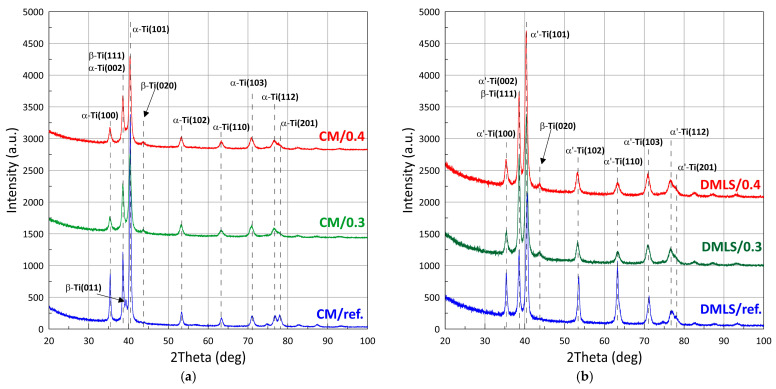
X-ray diffraction patterns of: (**a**) Wrought bar; (**b**) DMLS printed.

XRD patterns with designated peaks of Ti-6Al-4V phases are shown in Figure 8. The proportion of the individual phases as well as the size of the crystallites was identified using the Rietveld refinement and full width at half maximum calculations (Scherrer formula), Table 4 and Table 5 respectively.

Comparing the phase composition of Ti-6Al-4V made conventionally versus additively, it can be seen that the phase composition for the CM sample is α + β, while for the additive sample it is α’+ β. Significantly more α-phase is contained in the conventionally made sample and this is as high as 89%, compared to the AM sample which contains 78%. After the shot peening process, the proportion of the β-phase increases to 24–28%. Lavrys in his work [51] pointed out the positive effect on corrosion properties of β-phase growth during heat treatment performed on Ti-6Al-4V. The proportions of the phase after shot peening of the samples becomes similar regardless of the manufacturing method. The difference for CM/0.3 versus AM/0.3 samples is approximately 3%. And for CM/0.4 versus AM/0.4, it is statistically imperceptible at only about 1%.

Analysis of the peak FWHM values indicated significant grain refinement after post-processing treatment. The grain size decreased by a maximum of 51.3% for the DMLS printed sample and by 59.2% for the wrought sample. For these samples, the grain sizes oscillated between 9–9.5 nm. After the printing process, we are dealing with columnar grains, growing in the direction of heat dissipation [52]. Luo et al. [53] emphasized the critical importance of decreasing the grain size for improvement of corrosion resistance of Ti-6Al-4V. Ralston’s et al. [54] characterized the relationship between grain size and the corrosion rate of metals, pointing out an analogy with the Hall-Petch relationship applied to yield behaviour of a material.

In this work residual stresses were not analyzed, however, in our publication on the effect of shot peening on cavitation resistance [55], we obtained significant residual compressive stresses for a steel shot peened surface with a value of σ = −0.918 GPa. Such a value was obtained at a peening pressure of 0.3 MPa. Compressive residual stresses can enhance corrosion resistance, especially by delaying crack propagation and improving the integrity of the passive film, as demonstrated by Cruz et al. [56]. For SLMed 316L steel.

#### 3.2.2. Roughness and Hardness Measurements with SEM Analysis

**Figure 9:**
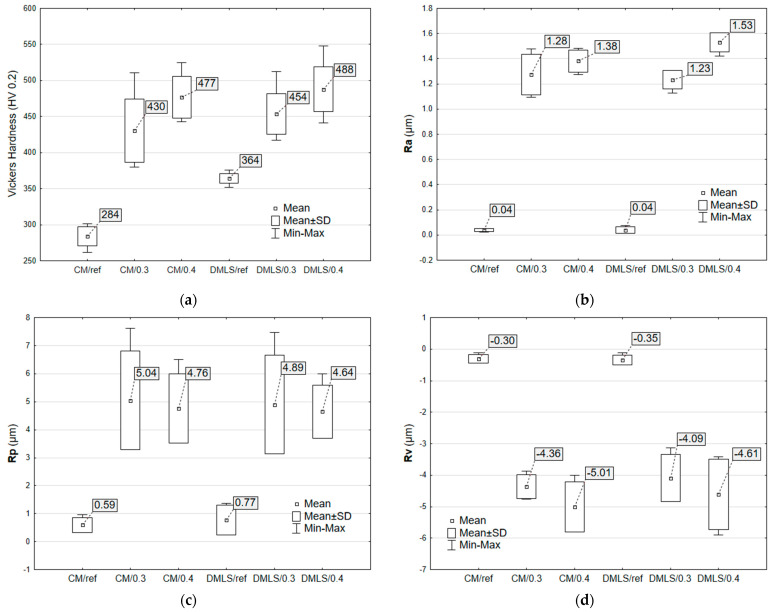
Surface characterization and mechanical properties of CM and DMLS Ti-6Al-4V samples: (**a**) Vickers hardness (HV 0.2); (**b**) Average surface roughness Ra; (**c**) Maximum profile peak height Rp; (**d**) Maximum profile valley depth (Rv).

Analysis of Figure 9a shows that changes in hardness were obtained for all modified samples. The greatest strengthening was obtained for the conventional samples and reached 68% for CM/0.4. This is due to the formation of a hard martensitic phase in the structure of the printed samples after the fabrication process, which resulted in a maximum increase in hardness of 34% for DMLS/0.4. The printed samples achieved higher hardness values than its conventional counterparts.

Roughness measurements of surface topographies revealed that shot peening forms uniformly distributed peaks and valleys on the surface due to the plastic deformation caused by the repeated impact of steel shots. The obtained results of maximum peak height R_p_ and maximum valley depth R_v_ did not exceed an average of 5.04 µm (Figure 9d) and 5.01 µm (Figure 9c), respectively. According to the arithmetical mean height R_a_ (Figure 9b) extracted from the shot-peened surface, 1.53 µm-high peaks and valley formed after shot peening, compared to the unpeened sample with a mirror-polished surface (0.04 µm arithmetical surface roughness).

**Figure 10:**
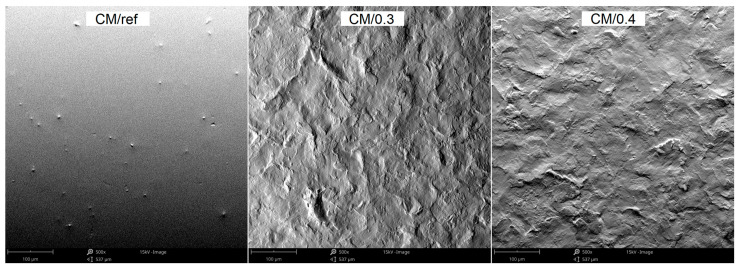
The morphology of CM specimens surfaces before and after peening with different pressure, SEM.

**Figure 11:**
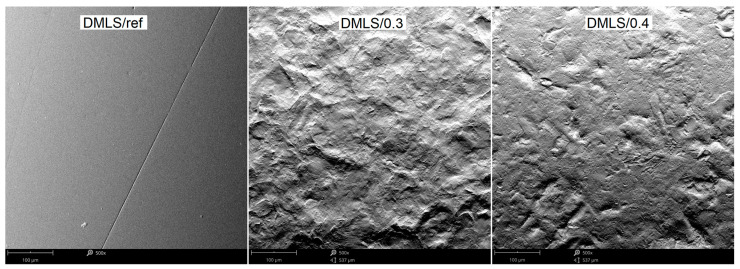
The morphology of DMLS specimens surfaces before and after peening with different pressure, SEM.

**Figure 12:**
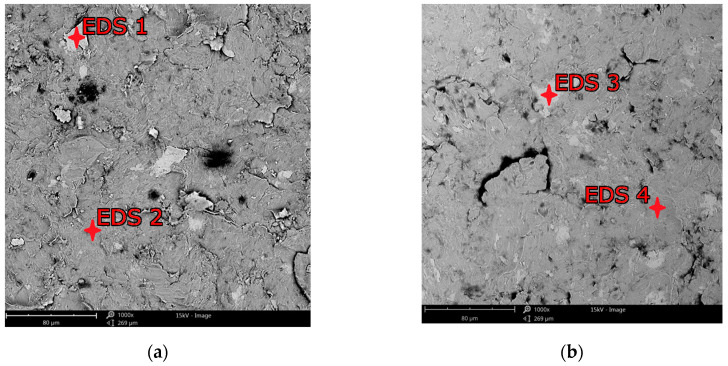
EDS examination spots for shot peened surfaces: (**a**) CM/0.3; (**b**) DMLS/0.3.

The surface profile results of the samples are confirmed by SEM images shown in Figure 10 and Figure 11. In these photos, it is evident that significant peaks relative to the profile can be observed in the case of 0.3 MPa shot peening. However, with the increase in the shot peening pressure, the depth of the deformed layer area deepens in form of the craters. This is due to severe plastic deformations on the surface of titanium alloy in the case of shot peening with higher pressure (0.4 MPa). Therefore, it can be seen that the surface roughness is slightly higher after the shot peening process with higher pressure. In Figure 12a,b there are visible remnants of embedded CrNi steel shot in the form of a difference in imaging. Using the BSD detector, bright reflections can be seen on both CM and DMLS samples. The analysis of the chemical composition based on the designated points for samples CM/0.3 and DMLS/0.3 is shown in Table 6.

**Figure 13:**
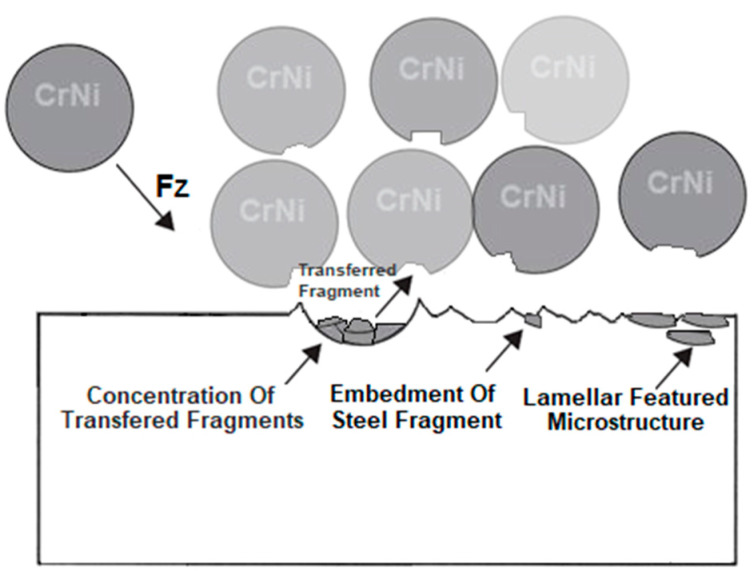
Scheme of the effects of surface layer modification by the shot peening treatment with CrNi steel shots based on Kameyama and Komotori’s model [57].

Residues of embedded shot were indicated by EDS spectral analysis and remain in the surface layer despite washing the samples in an ultrasonic cleaner. Such residues have a different electrochemical potential compared to the peened surface and the core of the material. It also provides more surface area for corrosive agents to attack. This is in line with Kameyama and Komotori’s model [57]. Žagar et al. [58] reports that contaminated surface of Aluminium Alloy 7075 with steel shots, in his case with S170 steel balls, is exposed to a heavier impact of the corrosion. This could lead to the higher susceptibility to the pitting corrosion [59]. In addition, in the case of cytotoxicity, release of cytotoxic elements associated with chromium-nickel steel shots contributes to the effect of metallosis in the long-term, which is undesirable. The cytotoxicity results cited in the introduction [35,36] are satisfactory and meet ISO 10993-5:2009 [60] standards however, it would be more preferable to remove these steel shot residues. A potential solution that could remove the unfavorable effects associated with embedded shot, while not removing the beneficial effects associated with the shot peening treatment could be a combination of shot peening and electropolishing processes [61], although this has not yet to been investigated. The characteristics of the lamellar shot formation process based on their model is shown in Figure 13.

### 3.3. Corrosion Behaviour

**Figure 14:**
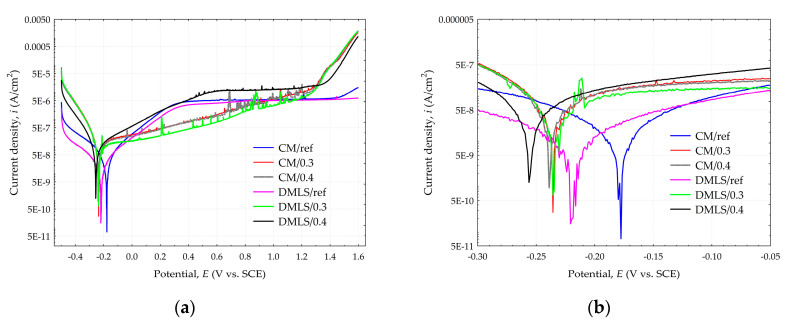
Tafel polarization curves for Ti-6Al-4V samples (**a**) Full view; (**b**) detailed view.

The potentiodynamic polarization curves of the conventionally and additively manufactured Ti-6Al-4V shown in Figure 14a and detailed in Figure 14b indicate that all treated and untreated samples exhibit a passivation process during the corrosion progression. It means the formation of a passivation film, which inhibits the corrosion of the material in the 0.9% NaCl medium solution. The shot peening treatment has an impact on both the anodic and cathodic polarization curve. For samples with a higher current density value, instability of formation of passivation film can be observed, resulting in poorer corrosion resistance [62]. Disturbances in the linear course of the curve are related to the occurrence of steel shot residues after the shot peening process. However, the stability of the passive layer formation cannot be necessarily be related to the pressure used in shot peening treatment as the cathodic curve for sample DMLS/0.4 is smoother than for sample DMLS/0.3 in the contrast to CM/0.4 and CM/0.3 curves. The results of the electrochemical parameters are presented in Table 7.

The results were analyzed statistically by the STATISTICA v. 13 Software (StaftSoft, Poland, Kraków) and have been subjected to the One-Way analysis of variance (ANOVA) and post hoc multiple comparisons on a basis of Tukey’s HSD tests. All parameters were considered statistically significantly different if *p* values were less than 0.05. The ANOVA test showed a statistically significant difference in the obtained electrochemical polarization results as the *p*-values were below 0.05.

On the basis of the current density value i_corr_, the corrosion rate CR was calculated. The calculation of CR was based on the ASTM Standard G 102-89 [63,64], according to the formula:

(3) $$CR=K\frac{i_{corr}}{\rho }EW,$$

where:

CR—corrosion rate [mm·yr^−1^], i_corr_—Current density of the corrosion [μA·cm^−2^], K—Reaction rate constant = 3.27 × 10^−3^ [mm·g·μA^−1^·cm^−1^·yr^−1^], ρ—density in [g·cm^−3^] = 4.4 g·cm^−3^ for Ti6Al4V was used, EW—equivalent weight.

The equivalent weight (EW) values were calculated from the formula:

(4) $$EW=\frac{1}{\Sigma \frac{n_{i}\cdot f_{i}}{M_{I}}},$$

where:

n_i_—valence of the i-th element in the titanium Ti6Al4V alloy, f_i_—mass fraction of the i-th element in the Ti6Al4V titanium alloy, M_i_—the atomic weight of the i-th element in the Ti6Al4V titanium alloy.

Calculations of the corrosion rate EW included only those components whose content in the alloy is not less than 1% wt. Calculated equivalent weight (EW) for CM samples was 11.90 and for DMLS samples 11.89, which corresponds to the literature data [65].

The corrosion rate CR with current density value i_corr_ and corrosion potential E_corr_ can be related to the value of the manufacturing method and the applied pressure. CM samples show slightly better properties than their DMLS sample alternatives. However, the applied peening pressure has a greater influence than the manufacturing method. This is closely related to the surface roughness. The polished reference surfaces had the lowest roughness and therefore the CM/ref as well as the DMLS/ref samples obtained the lowest corrosion current density values, which is 0.041 and 0.070 µA/cm^2^, respectively. The corrosion potential value was also the lowest for these samples and was −175.2 and −220.3 mV for CM/ref and DMLS/ref, respectively. The corrosion current density of the specimens subjected to 0.3 MPa pressure is higher and is 0.108 and 0.143 µA/cm^2^ for CM/0.3 and DMLS/0.3 specimens respectively. In this case, the corrosion potential value for sample CM/0.3 was significantly lower than the reference surface, at −233.6 mV. A similar value was obtained for the DMLS/0.3 sample, which was −235.9 mV. The worst corrosion resistance properties were obtained from samples peened with a pressure of 0.4 MPa. For samples CM/0.4 and DMLS/0.4 they were 0.172 µA/cm^2^; −239.1 mV and 0.217 µA/cm^2^, −259.9 mV, respectively.

The lowest corrosion rate was 3.63 × 10^−4^ mm·year^−1^ for CM/ref samples, which is about six times lower than DMLS/0.4 sample for which it was 19.18 × 10^−4^ mm·year^−1^. However, samples made conventionally were characterized by a higher standard deviation. For current density, relative standard deviation (RSD) for the CM/ref sample was as high as 31.7%, while for the DMLS/ref sample it was half of that, at 14.7%. For the peened samples, the RSD was below 10%, except for sample CM/0.3, for which it was 20.4%. For corrosion potential, the RSD for all samples was below 10%

Surface roughness is one of the decisive factors influencing the corrosion behavior of the materials. As the surface roughness decreases, the self-corrosion potential shifts in a positive direction, accompanied by a decrease in the corrosion current density [66], which can positively affect the corrosion rate of the material, while inhibiting pitting corrosion on the material surface [67]. Zhao et al. investigated the corrosion behavior of SiC/Ti6Al4V titanium matrix composites produced by SLM, where they indicated lower surface development together with low surface energy as the reason for obtaining low corrosion rate [68]. Wang also emphasized the importance of considering surface development when using Ti-6Al-4V titanium alloy in aggressive environments [69]. The roughness factor is much more significant than other factors favorably affecting the corrosion mechanism, such as grain refinement, an increase in the proportion of beta phase, or the development of compressive stresses discussed in Section 3.2.1.

**Figure 15:**
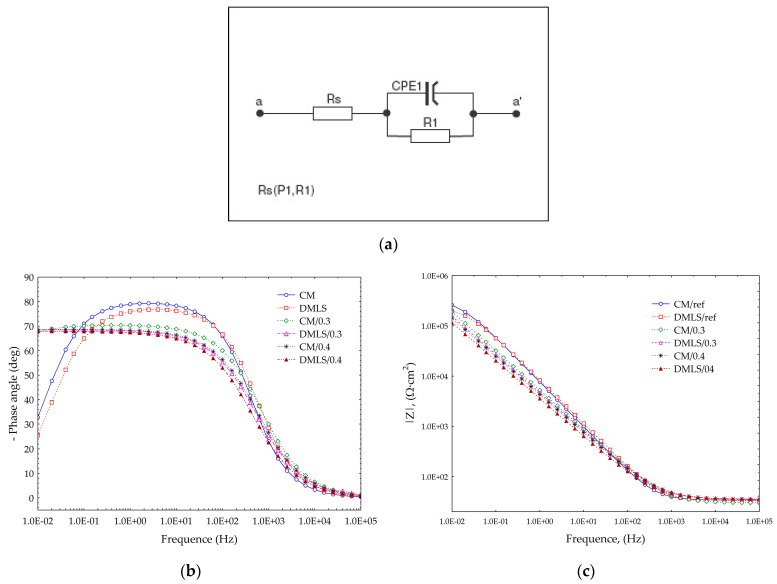
Effect of shot peening on the EIS of the Ti-6Al-4V titanium alloy in 0.9% NaCL (**a**) Electrical equivalent circuit; (**b**) Bode diagram, Phase angle; (**c**) Bode diagram, Impedance.

Given the type of electrical equivalent circuit used by Huang [28] or Toptan [70], the circuit shown in Figure 15a was used to simulate the non-ideal behavior of the capacitor associated with the passive oxide layer together with the electrolyte resistance (R_s_) and charge transfer resistance (R_1_). The results obtained during the EIS tests are shown in Table 8 and the Bode plot characteristics are illustrated in Figure 15b,c.

The simulated values included in Table 8 show that the highest values of R_1_ polarization resistance of the passive films was achieved for peened samples with 0.3 MPa pressure. This means that the passive layer formed on the surface of samples treated with 0.3 MPa has a higher protective capacity and better corrosion resistance. An increase in shot peening pressure resulted in a deterioration of the resistance value of R_1_. All samples that were subjected to the shot peening obtained better results than those which were untreated. On the basis of the acquired data, it is possible to calculate the equivalent of capacitance efficiency of the formed passive film C_eff_ on the tested specimens from Ti-6Al-4V. Equation (5) is the most commonly used for the calculations [71]:

(5) $$C_{eff}={Q_{dl}}^{\frac{1}{n}}{R_{1}}^{\frac{1-n}{n}},$$

However, much better approximation is the Brug [72] model (6), which was used to determine C_eff_ value shown in the Table 8 and is as follows:

(6) $$C_{eff}={Q_{dl}}^{\frac{1}{n}}{\frac{R_{1\;}\times R_{e}}{R_{1}+R_{e}}}^{\frac{1-n}{n}}$$

The average thickness of the surface layer production is closely correlated with the effective capacitance C_eff_ with the following Equation (7) [73]:

(7) $$C_{eff}=\frac{\varepsilon \varepsilon_{0}A}{\delta_{ox}},$$

where:

δ_ox_–thickness of the passive films, ε–dielectric constant of TiO_2_ passive film formed on Ti6Al4V = 95 according to the literature [74,75], ε_0_–dielectric constant of vacuum = 8.8542 × 10^−14^ (F·cm^−1^), A–exposed area of the sample (cm^2^)

The thickness of the passive films formed on the tested specimens from Ti-6Al-4V titanium alloy is inversely proportional to the capacitance efficiency of the formed passive film. In this regard, the average thickness of the passive layer after the shot peening achieve a significantly thicker layer than peened specimens. Depending on the type of manufacture and shot peening pressure, the differences in thickness between the reference and peened surfaces can reach up to almost 3 times. Additively manufactured samples produced a slightly thicker layer than conventional samples for the reference surface and peened with 0.4 MPa pressure. In the case of sample DMLS/0.3, the layer was thinner than for sample CM/0.3. When using model (4) and (5), Orazem et al. [76] showed that the difference between the actual layer state and the estimated state is in the range of −65% ÷ −68%. This is still a better result than with model (3) for which the estimated results were −89% ÷ −93%. In addition, using model (3) may lead to the misrepresentation that shot peening causes a decrease in the thickness of the passive layer instead of an increase. By introducing a correction to the appropriate adopted model, the resulting average thicknesses of the passive layer can be accurately estimated. The calculated results are shown in the Table 9.

The results obtained for corr. (4) correspond with the literature data [28,77]. Based on this literature data, it can be also stated that the passive layer obtained mainly consists of two fractions of TiO_2_ and Ti_2_O_3_. A denser and more corrosion-resistant passive layer is one that has a higher TiO_2_ content. XPS studies of Huang et al. [28] indicate a higher TiO_2_ content in the conventionally produced materials than in the manufactured additive. Huang determined the TiO_2_ content for the SLM Ti-6Al-4V sample to be 66.82%, and Ti_2_O_3_ to be 33.18%. In contrast, he determined the TiO_2_ content for the conventionally forged sample of Ti-6Al-4V to be higher as 67.89%, and Ti_2_O_3_ to be 31.50%. There was also 0.61% metallic Ti (Ti^0^) content on the surface. So, despite the 3D-printed products produced a thicker passive layer, its corrosion resistance was inferior.

An analysis based on Figure 15b of the Bode diagrams indicates that passive protective layer after the shot peening treatment is compact, closely adhering to the substrate. This is evidenced by the high angles of approximately 70° at low and medium frequencies. Reference surfaces achieve higher angles at mid frequencies reaching up to 80° and therefore better corrosion resistance, although at low frequencies they have much poorer resistance since their angle is approximately 30°. This is caused by the refinement of the grain after shot peening treatment [78]. In the case of AISI 304 stainless steel, the refinement of the grain after peening processes also resulted in improvement of corrosion behavior in low frequencies [79]. Such a dense and thick layer after shot peening performs well against pitting corrosion as demonstrated by Yoo [80] when he shot peened carbon steel and studied the corrosion behavior in the LiBr aqueous solution, as well as Huang [81] who indicated in his review a positive effect of the shot peening in aluminum alloys in inhibiting pitting corrosion.

From Figure 15b, it can be noted that there are no such statistically significant differences for the Z-modules obtained. All the impedance results obtained are high, above 10^5^. The best results were achieved by the reference surfaces; however, all samples had satisfactory values such as those of the materials used for medical implants.

## 4. Conclusions

The study concluded that:

- In the case of conventional samples, a two-phase α + β structure was obtained, in contrast to additively manufactured ones for which a martensitic structure α’ + β was obtained. The structure of all samples was dense and homogeneous.
- The porosity detected by the micro-CT is 0.0003%. A solid structure of DMLSed specimens from Ti-6Al-4V was obtained, which makes the effect of porosity on corrosion resistance negligible.
- The greatest strengthening effect was obtained for the conventional samples and reached 68% for CM/0.4. For printed samples, the maximum increase in hardness was 34% for DMLS/0.4 due to the formation of a hard martensitic phase after the fabrication process. The printed samples achieved higher hardness values than its conventional counterparts.
- Conventionally forged samples achieved slightly better results than their additive alternatives in terms of corrosion resistance. The lowest corrosion rate obtained was 3.63 × 10^−4^ mm·year^−1^ for CM/ref samples, when its additive counterpart DMLS/ref obtained 6.19 × 10^−4^ mm·year^−1^. The poorest properties were obtained for DMLS/0.4 sample as it was 19.18 × 10^−4^ mm·year^−1^. This is about six times higher corrosion rate than that for CM/ref.
- The applied peening pressure on pre-polished specimens has an enhanced influence on corrosion behavior than the manufacturing method. Increased pressure in range of 0.3–0.4 MPa in course of shot peening process with CrNi steel shots leads to increased surface roughness and as a result the deterioration of corrosion properties. Higher pressure increases the Ra and Rv parameters.
- Inclusions of CrNi steel shot particles have been observed in the surface layer after subjection to the shot peening process.
- Based on the values of the full width at half maximum (FWHM), the grain refinement after peening treatment was at least 54%. However, further increase in pressure resulted in a slight reduction in average grain size up to 11.7%.
- The shapes of the Bode plots of the impedance spectra are different depending on whether they were treated or not. The obtained values of phase angle were higher in low frequency range in case of surfaces subjected to shot peening due to grain refinement. In the mid frequencies range, values of phase angle of unmodified surfaces were higher as the surface roughness was lower than the roughness of peened samples.
- In low frequency range, all tested surfaces are characterized by high impedance above 10^5^ Ω·cm^2^ which points to suitable corrosion resistance in 0.9% NaCl solution for medical implants.
- All samples developed a compact and closely adhering to the substrate passive protective layer after the shot peening treatment.

## Figures and Tables

**Table 1:** Comparison of chemical composition declared by manufacturers (annealed bar DAIDO Steel Co., Ltd. (USA, IL, Schaumburg) and DMLS printed sample EOS GmbH (Krailling, Germany).

Element [Mass %]	Grade 5	
	DAIDO Steel (Bar)	EOS GmbH (Powder)
Al	5.5–6.75	5–6.75
V	3.5–4.5	3.5–4.5
Fe	≤0.40	≤0.30
O	≤0.20	≤0.20
C	≤0.08	≤0.08
N	≤0.05	≤0.05
H	≤0.0125	≤0.0015
Ti	Bal.	Bal.

**Table 2:** Parameters of CrNi shots which were used in the shot peening treatment.

Shot	Shot Parameters			
	Average Size (μm)	Grain Shape	Typical Chemical Composition (%)	Hardness
Steel shot-CrNi	400–900	Spherical	Cr: 16–20; Ni: 7–9; Si: 1.8–2.2 Mn: 0.7–1.2; C: 0.05–0.2; Fe: Bal	235 HV

**Table 3:** Notations of the specimens used in the experiment.

Manufacturing Process	Specimen Notation	Peening Pressure (MPa)	Peening Time (s)
Conventional (Annealed wrought)	CM/ref	unpeened	
	CM/0.3	0.3	120 s
	CM/0.4	0.4	
Additive Manufacturing (DMLS)	DMLS/ref	unpeened	
	DMLS/0.3	0.3	120 s
	DMLS/0.4	0.4	

**Table 4:** The proportion of the amount of individual phases in Ti-6Al-4V titanium alloy.

Sample No.	α-Ti (%)/α′-Ti (%)	β-Ti (%)
CM/ref	89.0	11.0
CM/0.3	76.0	24.0
CM/0.4	73.0	27.0
DMLS/ref	78.0	22.0
DMLS/0.3	73.0	27.0
DMLS/0.4	72.0	28.0

**Table 5:** Average size of Ti-6Al-4V crystallites.

Sample No.	Grain Size (nm)	Sample No.	Grain Size (nm)
DMLS/ref	19.3	CM/ref	22.3
DMLS/0.3	9.6	CM/0.3	10.3
DMLS/0.4	9.4	CM/0.4	9.1

**Table 6:** EDS analysis of weight contribution to chemical composition of Ti-6Al-4V individual elements for conventional and additive samples.

Element	CM/0.3		DMLS/0.3	
	EDS 1	EDS 2	EDS 3	EDS 4
	Weight Conc. [%]			
Fe	44.38	2.75	39.91	2.44
Ti	30.30	70.33	25.03	50.68
O	5.50	18.37	16.74	39.05
Cr	13.43	-	10.14	-
Ni	3.10	-	4.86	-
Al	2.72	6.35	2.52	6.21
V	0.58	2.20	0.79	1.61

**Table 7:** Electrochemical parameters for the Ti-6Al-4V specimens.

Sample No.	i_corr_	E_corr_	CR
	[µA·cm^−2^]	[mV]	×10^−4^ [mm·Year^−1^]
CM/ref	0.041 ± 0.013	−175.2 ± 11.3	3.63 ± 1.15
CM/0.3	0.108 ± 0.022	−233.6 ± 8.1	9.55 ± 1.95
CM/0.4	0.172 ± 0.017	−239.1 ± 7.0	15.21 ± 1.50
DMLS/ref	0.070 ± 0.005	−220.3 ± 13.1	6.19 ± 0.44
DMLS/0.3	0.143 ± 0.021	−235.9 ± 6.0	12.64 ± 1.86
DMLS/0.4	0.217 ± 0.014	−259.9 ± 5.4	19.18 ± 1.24

**Table 8:** Electrical equivalent circuit parameters of EIS spectra.

Sample No.	R_s_	R_1_	CPE_1_		C_eff_ ×10^−5^ F·cm^−2^
			Q_dl1_	n_1_	
	Ω·cm^2^	×10^5^ Ω·cm^2^	×10^−5^ Ω^−1^·S^n^·cm^−2^		
CM/ref	34.09	3.45	2.53	0.89	1.06
CM/0.3	29.95	33.56	4.52	0.79	0.78
CM/0.4	35.18	6.40	5.32	0.76	0.73
DMLS/ref	32.93	2.45	2.45	0.87	0.85
DMLS/0.3	35.36	31.90	5.78	0.76	0.82
DMLS/0.4	35.63	5.99	3.56	0.76	0.43

Q_dl_—constant phase element (CPE_1_) of passive film, n—exponent of Q_dl._

**Table 9:** Results of the calculation of the average thickness of the passive layer.

No. Sample	δ_0x_ (nm)			
	Approach (3)	Corr. (3)	Approach (4)	Corr. (4)
CM/ref	1.3	14.1	4.0	12.4
CM/0.3	0.2	2.7	5.4	16.8
CM/0.4	0.3	2.9	5.7	18.0
DMLS/ref	1.3	14.6	5.0	15.6
DMLS/0.3	0.1	1.6	5.1	16.1
DMLS/0.4	0.4	5.0	9.7	30.3

## Data Availability

The raw data supporting the conclusions of this article will be made available by the authors on request.
